# Indents

**Published:** 1909-08-15

**Authors:** 


### INDENTS.

☞Brief Articles of Technical or Scientific Value will receive notice and comment. Contributions invited.

*To Relieve Pulpitis.* If you want to get a patient promptly relieved of pulpitis, counter-irritate the gum tissue, over the offending tooth, and give internally 1-100 grain of nitroglycerin, in tablet form. There is nothing like nitroglycerin to relieve the blood pressure, and its effect is very rapid. Do not give the patient any of the tablets to take home. One tablet will be sufficient to get results. This treatment is especially indicated in cases where arsenic has been applied, and the patient returns with a severe throbbing tooth-ache. The relief is almost instantaneous.—W. H. Tweedle, in *Review.*

*Quick Method of Replacing a Tooth on a Vulcanite Plate.* Dovetail the plate in the place slightly more than if rubber were to be used. Adjust tooth to plate and retain by a large piece of wet absorbent cotton, holding plate and all in the hand. Use a small piece of acolite sufficient to fill space with a slight excess. Heat a small soldering iron in the blowpipe flame to almost redness, touch the acolite, and it will flow to place and securely hold the tooth. Finish with burs and stones. The process takes about twenty minutes and it is impossible to remove the tooth without breaking.—E. Marshall Bush in *Review.*

*Strength of Cast Gold for Crowns.* In the use of cast gold in daily practice for three years I have found that gold, as we ordinarily cast it, is one of the most frail materials we can use for casting to the edges of a shell crown. Cast gold is not, and can not be made as strong as the ordinary plate gold. Cast iron is not as strong as wrought iron of the same thickness. A thin cast shell crown going over a properly tapered root, the shell being filled with soft cement and crowded to place, is exceedingly liable to split and is a very treacherous support for anything. It will not last because it has not the strength.—L. H. Arnold, in *Review*.

*A Platinum Famine Threatened.* The world is threatened with a platinum famine, so dispatches from Russia say. The price of this metal is rising rapidly. Platinum is chiefly mined in the Ural Mountains, and Russia supplies 95 per cent. of the entire world's production. Never before has the output of the mineral sunk to so low a figure; in 1898 only about 4 tons 16 hundred-weight was mined. The production has been falling off steadily since 1901, when it amounted to 6 tons 5 hundredweight.

The reason for this shrinkage is not because the platinum mines are becoming exhausted, but because the owners have formed a combination with the object of restricting the output and thus forcing the price up. For this same reason new borings have not been made for a considerable time.

Although a scarcity of platinum would not affect industry in general very much, it is of grave importance to the scientific world, and the hope is expressed by many scientists that platinum deposits may be discovered elsewhere, so that the production may no longer remain dependent on the plans of a small number of Russian mining magnates.—*New York Sun.*

*Casting Metal.* If there is any one detail in the mechanical process more important than another, I should say that it is the melting of the metal. There is a certain condition of a molten metal at which that respective metal will cast to the best advantage, *and it is not the boiling*

*point.* Any ordinary blow-pipe may be used that will, with the proper manipulation, melt the metal quickly with a strong blue flame; in no instance should a large smoky flame be used for it only tends to oxidize and spoil the working of the metal' Once the metal has been melted, one should not be too. anxious to cast, but should employ a small soft flame for a short time upon the already molten mass until it is in the proper condition to flow into a mold. Take, for instance, in the ordinary casting of zinc and lead for dies, etc., considerable care is taken that the molten metal is not bubbling from being too hot, and, on the other hand, not sluggish from being too cold before pouring. If the metal be in the proper condition one need not be in a great hurry to apply the pressure, for it will remain in a liquid form some considerable time.

In conclusion, simply because the molten metal is to be driven by force into the mold is not a sufficient reason for being careless about the melting of the metal, even in such simple castings as inlays, and yet expect an apparatus of any description to duplicate perfect castings under such adverse conditions.—Dr. Le Crow in *Brief*.

*Professionally Interested.* About forty-five years ago an elder brother of mine, after I had attended one course of lectures, advised me to go back to Chicago the next day and attend the meeting which was called to organize the Illinois State Dental Society. This was the best advice I ever received. It probably did more for me than any other one thing, because I got into the swim, and I have been there ever since.

The last speaker spoke of how difficult it is to get men to work. I think we will get men interested more by experimental work along the line of post-graduate studies than in any other way. Experimental work is always fascinating. I think the most stupid man living would become more or less fascinated with it.

As the last speaker has said, it is a difficult problem to get everybody interested. We will never do it, because there are a lot of men attempting to practice dentistry who ought not to be practising dentistry. You all know that. The whole thing centers upon the one thought of getting men aroused to earnestness. If we can get every practitioner in earnest, so that he will do the best he can, something can be accomplished. This post-graduate work ought to help us very materially, and I have no doubt it will. I want to encourage it more than any other one thing, as I am confident it will very much improve the Dental profession.—Dr. J. N. Crouse.

*Size of the Casting Sprue.* On account of the metal being melted in a crucible, directly over the opening or lead into the mould, instead of pouring the liquid mass into a runner—this lead or gate must be much smaller in diameter. If too large the metal is liable to sag into the same; if too small the construction offers too great an obstruction to the flow of the metal. With small castings, it is remarkable what a small gate opening the metal may be forced through.

I have been trying to determine by a series of experiments, not yet completed, the *size, length* and number of the gate wires necessary for the various castings in the different metals; for each metal allows of a different treatment. With aluminum or any of the lighter metals, most any size gate seems to answer the purpose; but as there is no tendency for these metals to sag a very large gate will be found most useful. The heavier metals in large castings require more care in the selection of the gate wires; with such metals as tin and Watts metal that melt at a low temperature and remain liquified for some considerable time, smaller gates may be used than with the same case in gold that solidifies quickly.

If attempting a large casting in gold through one lead, there is an influence towards using a large gate wire, thereby rendering a failure more possible. Hence I prefer to use two or more smaller gates leading to various parts of the mold to minimize uncertainties. Casting a metal into a mold in bulk, is quite different from that of casting the same quantity where it must spread for some distance from its entrance into the mold.

In short with the data to hand, I can but say that I adhere to the following details as near as possible until I satisfy myself more fully on this subject, *i. e.*, the heavier and the greater the bulk of the metal to be used with the exception of tin and Watts metal and similar metals and the thinner the object to be cast, the smaller the gate openings and the greater the number of the same radiating from a common centre to various parts of the mould and last, but by no means least, the flatter the crucible or surface upon which the metal is to be melted.—Dr. Le Cron in *Dental Brief*.

*Death from Misadventure.* Dr. Wynn Westcott held an inquest at Bethnal Green, on the body of Kate Garcia, aged 36, the wife of a compositor, of Baroness Road, Hackney Road.

The husband stated that on the 4th inst. his wife went to the Mildmay Mission Hospital, Bethnal Green, to have her teeth attended to, and next day had four taken out. Next day she complained of illness, and a doctor who was called in said she had influenza and pleurisy. She was admitted to the hospital again on Tuesday, and died on Friday last.

Dr. C. H. Corbett, of the Mildmay Mission Hospital, stated that the deceased woman was put under the influence of ethylchloride when the teeth were extracted. She suddenly ceased breathing, and efforts were at once made to restore her. These were successful, and she was kept in the hospital for three hours before being allowed to go home. On her re-admission it was thought she was suffering from pleurisy and pneumonia. She gradually got worse and died on Friday. At the post-mortem examination a bit of the root of a tooth which the patient had inhaled was found in the bronchus, and this had set up an abscess and septic pneumonia which was the cause of death.

The Coroner said this was a very unusual cause of death, which did not occur once in a million times. If a whole tooth was being taken out it was easily missed, but if only roots were being removed it was not quite so easy to be certain whether a bit was lost or not.

The jury returned a verdict of death by misadventure.—*British Dental Journal.*

*Report of Dental Inspector of Public Schools.* Dr. J. H. Wagner, dental inspector in the public schools of Elizabeth, N. J., submitted a highly interesting report to the Board of Education recently. Among the remarkable points brought out is a statement to the effect that only two per cent. of the children in one of the schools of the city use a tooth brush. The investigations of the inspector show that few of the pupils are free from serious defects of the teeth and he advised that an effort be made to bring about a general improvement in this direction. The inspector advanced the opinion that if the children could be brought to use a tooth brush and some kind of powder at least once a day about fifty per cent. of the defects would be avoided. The report is the most exhaustive so far presented by Dr. Wagner. It is as follows:

"During the month of February I examined 638 pupils of School No. 5, and found the mouths and teeth of 142 to be in fairly good condition. The teeth of the remaining 496 were defective either because of small cavities, or else the teeth were broken down to such an extent that extraction is necessary. Of these, 183 were children with temporary teeth, 313 had permanent teeth, and every one of these pupils had one or more of the permanent first molars decayed.

"I found 503 cavities in the first molars that can easily be filled, sixty-four that can be crowned, thirty-three that need to be extracted, thirty missing, which were already extracted. I also found the mouths of thirty to be in a very foul condition.

"About two per cent of the children use a tooth brush.

"In School No. 6, I examined 362 children, of which number I found that the teeth of ninety-one were in good condition, and that 271 have defective teeth. Of the latter, 167 have decayed first molars. There are five cases of irregularities, which in all probability will cause a deformity of the mouth. This is due to the retention of roots of decayed temporary teeth.

"In one case, that of a child of seven years, the root of a temporary molar lodged itself crosswise in the process and cut into the mucous membrane and buccinator muscle, thereby causing a multiplication of cells. The child could not open its mouth without having the sharp ragged edge cut into the flesh. In an adult, I venture to say, it would cause cancer.

"I also found 253 permanent first molars with cavities that can easily be filled, thirty-nine that can be crowned, fourteen needing extraction, and found thirty-six to be missing, that were already extracted.

"From this report you can easily see that the condition of the children's mouths and teeth in certain schools could hardly be any worse. The importance of a clean mouth in relation to the general health is so great that exaggeration is impossible. In the thirty cases I report to you as having foul mouths, I found the teeth covered with tartar, green stains; cup-shape cavities in the molars filled with particles of food eaten several days ago, in which pathogenic or disease-producing bacteria flourish in abundance; and a breath that makes its presence known.

"If there were a means of inducing every child to use a tooth brush and some sort of powder, at least mornings, fully fifty per cent. of the cases I met would not exist."—*Dental Brief.*

*Syphilis.* Syphilis, from the standpoint of the dentist, is undoubtedly the most important and practical disease of the mouth with which he has to contend. There is no class of medical practitioners who, in my opinion, are so much in danger of accidental infection with syphilis as dentists; and this is on account of the mucous patches. Mucous patches are extremely insidious, because they may cause almost no pain and there may be no other easily found evidence of syphilis. They are highly contagious and, it seems to me, the dentist cannot be too careful in guarding against the possibility of danger from this source. Of course, you have, also, chancres in the mouth or about the mouth to guard against, but in my experience chancres about the mouth, except on the lips, are uncommon. Chancres of the lips are not excessively uncommon in hospital practice, or in private practice. Usually, they are quite readily diagnosticated. There is one interesting point about chancres of the lips and mouth; and that is that they present a much uglier clinical picture, as a rule, than chancres about the genitals. A chancre about the lips is usually a lesion as large as a dime or larger, with firm induration and sharply defined neoplastic infilteration. It is also accompanied, as a rule, by marked lymphatic engorgement. You will get quite large tumors of the glands of the neck in chancres. The diagnosis from epitheliomas as a rule does not offer much difficulty and this is the only thing it is apt to be confused with. The lesions are more circumscribed; there is less ulceration; there is a different sort of induration; and there is the sudden development of enlargement of the glands. That is not the picture of epithelioma. However, chancres are sometimes diagnosticated and operated on as epitheliomas.

Mucous patches have been described fully by Dr. Gilmer, and I do not know of anything to add to his phase of the subject except this: When you are in doubt about the character of mucous patches or of ulcers on the lips or about the mouth, examine for the spirocheta pallida of Schaudinn. There seems to be left no room for doubt that the spirocheta pallida is the pathogenic organism of syphilis. This organism has a characteristic morphology, and it is easily found. It is not difficult to find, and you can, I think, with reasonable certainty make a positive diagnosis of specific disease now upon finding the spirocheta pallida.

Gummata occur about the mouth with great frequency, but I imagine they occur most frequently in a class of patients you do not see in private practice. I see many of them in public practice, but in private practice we don't see many of them. They occur in people who are not apt to pay much attention to the condition of the mouth. As to the infectious character of gummata, that is a point upon which I think there is some room for comment. It has been definitely established that occasionally gummata are infectious, but the part of the gumma that is infectious is the active spreading border. The degenerated center is not infectious. There is strong clinical proof of that fact, and we have now other proof; in the borders of gummata spirochetes have been found. The danger to the dentist however does not lie in the tertiary lesions of syphilis, but particularly in the mucous patches.—Dr. W. A. Pusey in *Review.*

*The Father of Dentistry.* Pierre Fauchard was born in Brittany toward the close of the XVII century, he was destined by his parents for the practice of surgery, but being prevented form doing this he became the disciple of Alexander Poteleret, surgeon in chief to the King's ships. Viau says that Fauchard had tried several mechanical pursuits, the practice of which proved to be not without value to him later on. In fact it was the preliminary manual training he was unconsciously receiving. He was fortunate in studying under Poteleret, for this able naval surgeon was experienced in oral troubles, especially in scorbutic disorders, which at that time were frequent on vessels making long cruises. So important did they consider this matter that at the beginning of the eighteenth century the port of Brest was provided with a surgeon-dentist and later, in 1730. When the great minister Colbert organized the French navy, he instituted a health service, which, let us be thankful, was not too strict in the entrance requirements, and Frauchard was able to join as assistant. His service in the marine corps did not continue long, for in 1700

he took up his residence in Angers, afterwards traveling from town to town to make a livelihood, visiting Tours, Rennes and Nantes at first dates. And we may say of him as was said of Chapin A. Harris more than a century later as he traveled from town to town in the west—that wherever he went the public estimation of dentistry was elevated and his own reputation was established. Although his fame increased and people came to seek him from the depths of Brittany, he determined to test his abilities on a larger scale, and in 1719 went to Paris, where at this time, and even earlier, as we have seen, there were not only the tooth-pullers of the Pont Neuf, but also dentists properly so called.

Indeed, Fauchard mentions the examination that aspiring dentists had to undergo as far back as the year 1700. It is not strange that this broad minded man met with success in Paris and quickly had for his friends not only the well known dentist, Carmeline, but also many surgeons and physicians. His great work, "Le Chirurgien-Dentiste," was published in 1728 in two volumes of over eight hundred pages, with forty full-page illustrations. To many of the present day this work would be a revelation, and it is much to be regretted that it was not translated into English at a time when the information it contained would have had practical value. It was translated into German and also passed through several editions in French.

"A careful reading of the subjects treated in Fauchard's work," says Dr. Wm. H. Trueman, "will show how fully and systematically he covered the whole ground of dental science and refutes the common idea that dentistry in his day consisted in tooth-pulling and tooth-replacing. In my judgment as an original and comprehensive exponent of dental science it had no rival in the English tongue until Harris' 'Principles and Practice' had reached its second or third edition."

After a long and honorable career this illustrious ancestor of ours died at his residence in the Rue des Grande Cordeliers on the 25th of May, 1759—the exact date has been a matter of some dispute.

Chapin A. Harris said of him: "He found the dental art a crude branch of mechanics, he left it a digested and systematic branch of the curative art. Though his own practice was far inferior in excellence to that of our day; though his instruments were crude, and the many appliances of his art very deficient in completeness and nicety of adaptation. yet, considering the circumstances under which he lived, Fauchard deserves to be affectionately remembered as a noble pioneer and sure founder of dental science. That his practice was crude was due to his times; that it was scientific and comparatively superior and successful was due to himself."

He was an inspiration and example to his countrymen, and should be so to all dentists, and I wish to place myself again on record as sincerely believing that the day will come when some public recognition of the obligations we are under to this great Frenchman will be made and that a statue of him will be erected in the city of Paris by the grateful contributions of the dentists of America.—Dr. McMannis in *Review*.

